# Supplementary material for: Tetraplex Fluorescent Microbead-Based Immunoassay for the Serodiagnosis of Newcastle Disease Virus and Avian Influenza Viruses in Poultry Sera
Source: Pathogens. 2022 Sep 17;11(9):1059. doi: 10.3390/pathogens11091059 (PMC9505202; doi:10.3390/pathogens11091059)
Supplement: Supplementary file 1 [file pathogens-11-01059-s001.zip › pathogens-1911160-supplementary.pdf]

Supplemental materials

Table S1. Positive and negative control sera for assay validation.

	HI			Commercial ELISA				Western Blotting			
Serum	NDV clone 30	AIV H5N3	AIV H7N7	IDEXX NDV NP	iD.Vet AIV NP	iD.Vet H5	iD.Vet H7	NDV rNP	IAV rNP	H5N3 rHA1	H7N7 rHA1
S82 (H5N1[a])	4	**>512**	4	neg.	**pos.**	**pos.**	neg.	-	**+**	**+**	-
S304 (H7N3[a])	2	<2	**>512**	neg.	**pos.**	neg.	**pos.**	-	**+**	-	**+**
S185 (ND-Ulster[1])	**>512**[b]	<2	<2	**pos.**	neg.	neg.	neg.	**+**	-	-	-
SPF serum	4	<2	4	neg.	neg.	neg.	neg.	-	-	-	-

[a] - Antigen used to raise the sera

[b] - Bold-face values indicate specifically positive reactions. Western blotting reactions are also depicted in Figure 2.

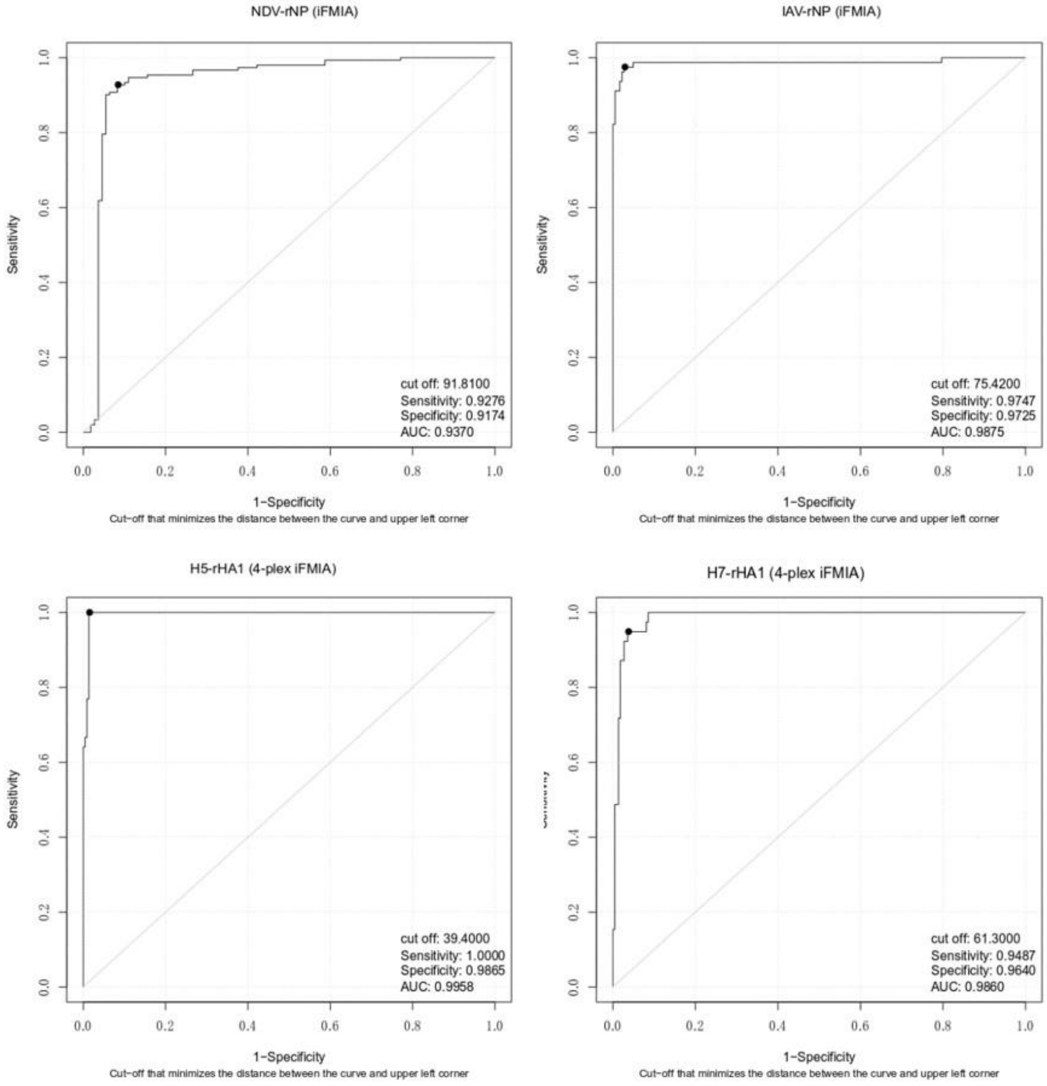

Figure S1. ROC analyses of the 4plex iFMIA for performance characteristics in sera from experimentally infected and/or vaccinated galliform poultry. ROC curves of the 4plex iFMIA reactivity to 4 recombinant proteins of NDV and AIV were compared with those of HI assay (n = 257).
